# Inviting eating disorder patients to discuss the academic literature: a model program for psychoeducation

**DOI:** 10.1186/s40337-017-0178-7

**Published:** 2017-11-03

**Authors:** Lauren Belak, Tara Deliberto, Matthew Shear, Sean Kerrigan, Evelyn Attia

**Affiliations:** 1 Department of Psychiatry, Weill Cornell Medical College, New York-Presbyterian Hospital, 21 Bloomingdale Road, White Plains, NY 10605 USA; 2 Department of Psychiatry, Columbia University Medical Center, 1051 Riverside Drive, New York, NY 10032 USA

**Keywords:** Anorexia nervosa, Bulimia nervosa, Psychoeducation, Novel intervention

## Abstract

**Background:**

Psychoeducation initiatives in which patients read primary scientific literature have not yet been studied as a treatment intervention for eating disorders. In this paper, we discuss and evaluate the acceptability of a novel psychoeducational journal club for individuals with anorexia and bulimia nervosa in inpatient and partial hospitalization program settings. Primary literature about eating disorders is presented and discussed with patients. By presenting scientifically-supported information, our “Psychoeducational Research Group” is designed to help patients restructure disordered thoughts and encourage adherence to evidence-based treatment.

**Methods:**

Using a Likert scale questionnaire (0 = not at all; 5 = very much), participants provided ratings for how much they liked the group and felt that it helped them across several domains.

**Results:**

Average scores from 33 participants (26 inpatient, 7 partial hospital patients) indicated they would recommend this group to others receiving eating disorder treatment (4.8 ± 0.6). Scores also suggested patients’ likeability (4.6 ± 0.8), benefit regarding challenging eating disorder thoughts (4.1 ± 1.1), improved motivation for eating behavior change (4.0 ± 1.0) and completion of prescribed nutritional plan (3.6 ± 1.0), and usefulness in working towards treatment goals (4.2 ± 0.9) associated with group participation.

**Conclusions:**

Preliminary findings support the acceptability of this psychoeducational group and that it may serve as a useful adjunct to larger evidence-based programming across eating disorder treatment settings.

## Plain English summary

Eating disorders are life-threatening mental illnesses that are notoriously challenging to treat. Because rates of mortality and relapse are significantly elevated in individuals with anorexia nervosa or bulimia nervosa, efforts to help improve existing treatment approaches are greatly needed. Currently, psychoeducation, or the provision of knowledge about psychiatric illnesses, is offered to patients with eating disorders as a core component of care. Here we describe and evaluate a “Psychoeducational Research Group,” an innovative form of psychoeducation, in which scientific articles about eating disorders are presented and discussed with patients. This research was conducted in inpatient and partial hospitalization settings with patients who have a diagnosis of anorexia or bulimia nervosa. Group participants were asked to complete an anonymous questionnaire assessing how much they liked the group and felt that it helped them with challenging eating disorder thoughts, improving motivation for eating behavior change, and working towards treatment goals. Average scores suggested the group’s acceptability and self-reported utility among such patients. This “Psychoeducational Research Group” may serve as a useful adjunct to larger evidence-based programming across eating disorder treatment settings.

## Background

Eating disorders are prevalent and life-threatening psychiatric illnesses. In the United States, 1.5% of women are diagnosed with bulimia nervosa (BN) and 0.9% with anorexia nervosa (AN) at some point during their lifetime [1]. Standardized mortality ratios are as high as 5.86 for AN and 1.93 for BN [2], and as many as 30–50% of patients with AN or BN relapse after reaching partial remission [3, 4]. Given the severity and chronicity of these disorders, improvements to existing treatment approaches are sorely needed.

Reluctance to pursue or complete treatment among patients with eating disorders is common [5–8] and may help explain the reduced efficacy of current modalities. For instance, some patients may view engaging in eating disorder behaviors as a way to reduce anxiety or gain control, others may have limited motivation for change, and still others may be wary of therapeutic interventions [9, 10]. Further, many people with eating disorders have limited insight into their illness’s severity, and/or have anosognosia, a severe and unintentional unawareness or denial of their illness [11]. Because of this inability to perceive neither psychological nor medical problems, many with eating disorders do not seek treatment [10].

While treating anorexia and bulimia nervosa can be challenging, cognitive behavioral therapies have been effective in the treatment of adult patients with BN, and demonstrate some efficacy for patients with AN [12–15]. A core component of these approaches is to offer patients psychoeducation, or information about their psychiatric illnesses, in order to help them modify the maladaptive cognitions and behaviors that maintain their disorders [12, 14]. Psychoeducational initiatives have been beneficial in the treatment of other psychiatric illnesses, such as depression [16], and have shown promise among eating disorder populations. For instance, Andrewes et al. discovered that outpatients with AN or BN who received a computer-presented health education package had significant improvements in their knowledge and attitudes towards their disorder when compared to the placebo group [17]. Additionally, Tatham et al. found four sessions of pretreatment psychoeducation helped to reduce bulimic patients’ unhealthy eating attitudes [18]. Structured treatments, such as inpatient and partial hospitalization programs, provide a variety of group therapies and activities that may incorporate psychoeducational elements; however, such psychoeducation is often presented as a broader summary of relevant medical information or research. To our knowledge, formal psychoeducation that exposes patients to primary literature first-hand has not been studied in these settings.

Here we sought to describe and evaluate a psychoeducational journal club group in which eating disorder related research articles are discussed with inpatients and outpatients with AN or BN. Topics such as the medical complications of eating disorders, the resolution of these complications with treatment, and the predictors of treatment outcomes are presented and openly talked about with patients. Journal clubs are used in academic teaching settings to help graduate and professional students become more familiar with advanced literature in their particular field. Our Psychoeducational Research Group is analogous, as patients who are directly affected by eating disorders should have access to and be educated in research pertinent to their illnesses. It is our understanding that a formal, ongoing group of this nature has never been offered nor evaluated in the treatment of eating disorders.

In this paper, we discuss a preliminary and exploratory investigation of this novel intervention offered in the context of multi-modal structured programs for the treatment of severe eating disorders. We hypothesized that patients would consider this group acceptable, and find that it is helpful for challenging eating disorder thoughts, improving motivation for eating behavior change, and working towards treatment goals.

## Method

This project was reviewed and accepted by the Office of Research Integrity of the Institutional Review Board at Weill Cornell Medical College.

### Procedures

#### Participants

Group participants were patients who met DSM5 criteria for either AN or BN [19]. Participants were receiving treatment at either the inpatient or partial hospitalization program for eating disorders at New York-Presbyterian Hospital. A total of 40 adults were admitted to the inpatient unit during the study period and 26 participated in this Psychoeducational Research Group study. Of the ten partial hospitalization patients admitted to the program, seven participated. During the study period, 93% of (*n* = 37) patients admitted to the inpatient unit identified as female and 7% (*n* = 3) identified as male. All ten patients admitted to the partial hospitalization program identified as female at the time of the study. Those who had been diagnosed by the clinical team with a severe cognitive impairment or other severe psychiatric illness (i.e. psychotic disorder) were excluded from this study.

#### Group design

Weekly, hour-long Psychoeducational Research Group sessions are scheduled as part of the specialized eating disorder inpatient and partial hospitalization programs at NewYork-Presbyterian Hospital in White Plains, New York. Programming at both the inpatient and partial hospitalization levels of care is aimed at decreasing eating disordered behavior through behavioral management, nutritional rehabilitation, and cognitive behavioral therapy. On average, approximately 13% of patients receiving treatment on the inpatient unit have been involuntarily admitted. The average length of stay for patients who are receiving treatment for an eating disorder on the inpatient unit is approximately 12.6 ± 8.8 days and 31.6 ± 27.6 days for the partial hospitalization program. At both levels of care, enrollment in the Psychoeducational Research Group is rolling, with participants joining on admission and leaving upon discharge.

At the beginning of each Psychoeducational Research Group, patients are provided with copies of a published research article (See Table 1 for examples). The article is selected by research and clinical staff based on the most common concerns expressed by the patients during rounds over the prior week. Patients are given at least 15 min to review the article and up to 30 min for longer and/or more challenging material. They are reminded that primary literature can be challenging to read and digest, and are encouraged to do their best. They are assured that the information will be clarified and their questions will be answered during a subsequent open discussion of the paper. While patients are reading, a group leader writes out bullet points of the article’s research question, method, and study results on a demonstration board. The group leader then presents the research to the patients, directs their attention to the article’s tables and graphs, and fields questions from the audience. The group is then open to discussion during which patients may comment, ask further questions, and raise concerns that the leader can address.

**Table 1:** A list of clinical topics and corresponding research articles presented during the Psychoeducational Research Group, in no particular order

Medical Complications of Anorexia and Bulimia Nervosa	• Minnesota starvation experiment [Kalm & Semba, 2005] • Cardiac complications of eating disorders [Casiero & Frishman, 2006] • Medical complications associated with purging [Forney, Buchman-Schmitt, Keel & Frank 2016] • Osteoporosis and bone loss in eating disorders [Zipfel et al., 2001; Olmos et al., 2010] • Endocrine abnormalities [Misra & Klibanski, 2014] • Cerebral atrophy in anorexia nervosa [Roberto et al., 2011] • Neuropsychological functioning deficits in the context of starvation [Laessle et al., 1990; Moser et al., 2003] • Delayed gastrointestinal transit in anorexia nervosa and bulimia nervosa [Kamal et al., 1991] • Reduced interoceptive awareness in patients with AN [Pollatos et al., 2008] • Negative effects on mood when dietary fat intake is reduced [Wells et al., 1998] • Electrolyte and acid-base abnormalities associated with purging behaviors [Mehler & Walsh., 2016] • Medical complications of laxative abuse [Mitchell & Boutacoff., 1986]
Improvement in Medical Complications Over the Course of Treatment	• Improvement in sleep patterns with weight restoration for patients with AN [Lacey et al., 1976] • Reversal of structural brain abnormalities after long-term recovery from AN and BN [Wagner et al., 2006] • Normalization of delayed gastric emptying during refeeding in patients with AN [Robinson et al., 1988; Rigaud et al., 1988] • Improvement in levels of depression and anxiety during re-nourishment patients with ED’s [Sala et al., 2011] • Neuropsychological functioning deficits resolve with inpatient treatment [Moser et al., 2003]
Efficacy of Treatment	• Efficacy of exposure therapy for improving eating behavior [Steinglass et al., 2014] • Improvement of body image disturbances during weight recovery in AN [Sala et al., 2012] • The brain is not static: how neuroplasticity underlies recovery [Kays et al., 2012] • Improvements in quality of life with symptom remission and recovery [Mitchison et al., 2016]
Predictors of Treatment Outcome	• Role of motivation and readiness for change on reduction in eating disorder symptoms [Ålgars et al., 2015;, Bewell & Carter, 2008] • Baseline predictors of better outcomes and time to remission [Vall & Wade, 2016; Clausen, 2008] • Diet energy density and diet variety during treatment are predictors of long-term treatment outcome [Schebendach et al., 2008]
Rationale Behind Treatment Approach	• Importance of consistent intake in breaking the restrict, binge, purge cycle [Ellison et al., 2016] • A literature review of nutritional rehabilitation in anorexia nervosa and its implications for treatment [Marzola et al., 2013] • How increased resting energy expenditure necessitates caloric increases for appropriate weight gain in patients with AN [Obarzanek et al., 1994; Van wymelbeke et al., 2004] • Short and long term efficacy of CBT in the treatment of bulimia nervosa [Bailer et al.,2004] • Efficacy of CBT for the management of anxiety, mood, personality and eating disorders [Hoffman et al., 2012]

Over the course of a patient’s enrollment in Psychoeducational Research Group, the group leader seeks to achieve four primary goals. First, the leader provides patients with objective facts to help them challenge cognitive distortions (e.g., that consistent intake or consumption of fat will lead to immediate body changes) [20]. Second, the leader works to improve patients’ insight into the gravity of their conditions and combat anosognosia by explaining *how* eating disorders can disturb organ systems. Third, the leader elucidates with concrete evidence how many of the complications associated with eating disorders (e.g. depression, gastroparesis, body image disturbances, cognitive decline etc.,) can resolve with treatment. Finally, by discussing the determinants of long-term treatment outcome, the leader helps patients identify their own obstacles in recovery in order to promote their future success in treatment.

#### Group assessment

Prior to admission to the inpatient or partial hospitalization at NewYork-Presbyterian Hospital, patients with AN or BN were informed by clinical staff that they may voluntarily participate in anonymous program evaluation questionnaires over the course of their hospitalization. As part of these assessments, eligible participants who attended the Psychoeducational Research Group for the first time were asked if they would be willing to complete the “Psychoeducational Group Questionnaire,” which assesses the favorability and usefulness of the group. Participants completed the survey following this first experience and within the first week of their admission.

The “Psychoeducational Group Questionnaire” was written for the purposes of this study. The seven-question assessment was designed de novo specifically to aid the journal club leader in developing this group and investigating its impact. Using a Likert scale (0 = not at all; 5 = very much), the measure assesses likeability, utility in challenging negative eating disorder thoughts and working towards treatment goals, and level of motivation to change eating disorder behaviors and complete prescribed meal plan associated with group participation (see Table 2). The questionnaire also assesses whether patients would recommend the group to other patients receiving treatment for an eating disorder, and open-endedly how the group could be improved. All questionnaires were collected devoid of identifiable information.

**Table 2:** Average scores of patients’ responses to Psychoeducational Research Group feedback survey

Assessment Question	Average Score Response (*N* = 33)
How much do you like this group?	4.6 ± 0.8
How much does this group help you in challenging negative eating disorder thoughts?	4.1 ± 1.1
How much does this group motivate you to change eating disorder behaviors?	4.0 ± 1.0
How much does this group motivate you to complete your caloric prescription?	3.6 ± 1.0
How useful has this group been in helping you work towards your treatment goals?	4.2 ± 0.9
Would you recommend this group to other patients receiving treatment for an eating disorder?	4.8 ± 0.6

Responses were assessed using a Likert scale (0 = not at all; 5 = very much)

Press Ganey patient satisfaction survey scores were also assessed for the designated study period to serve as a basis of comparison for patients’ experience with the Psychoeducational Group in particular. Over the past 30 years, Press Ganey has served as a strategic partner to nearly 10,000 health care organizations; offering patient experience surveys that combine required Hospital Consumer Assessment of Healthcare Providers and Systems questions with scientifically-developed patient-centered inquires. These assessments provide a comprehensive view of the patient experience to help health care organizations improve patient satisfaction across all levels of care. The Press Ganey surveys that measure patients’ general satisfaction with group programming on the inpatient unit and group therapy in the partial hospitalization program are provided here.

## Results

While this group was established in the spring of 2015, its evaluation was conducted with 33 consecutively admitted group participants (26 inpatient, 7 partial hospital patients) who were admitted between February 2nd 2017 and April 26th 2017 to either the inpatient or partial hospitalization programs for eating disorders at NewYork-Presbyterian Hospital. Results of participants’ responses to the “Psychoeducational Group Questionnaire” are depicted in Table 2.

Because no identifiable information was collected from this sample of 33 Psychoeducational Research Group participants, ratings provided on the Psychoeducational Group Questionnaire could not be matched with Press Ganey surveys. However, to gauge general satisfaction among patients treated in the inpatient partial hospitalization settings, Press Ganey survey scores were collected from 31 inpatients and ten partial hospitalization program patients admitted during the study period, between February 2017 and April 2017. Notably, not all patients who completed Press Ganey surveys participated in this study. Further, not all patients admitted to the inpatient unit completed Press Ganey surveys. A total of 40 patients were admitted to the inpatient unit during the study period and 31 completed Press Ganey surveys. All ten partial hospitalization patients admitted to the program during the study period completed Press Ganey surveys. On a scale of 0 to 100, inpatients’ mean satisfaction with their overall treatment experience was 74.3 ± 7.8, and 72.6 ± 9.3 for the inpatient program’s activities. Partial hospitalization patients’ average satisfaction rating of the program’s group therapy was 77.5 ± 24.9. Extrapolating from the five-point scale from the first item of Table 2 regarding patient satisfaction with the Psycheducational Research Group, the average satisfaction score was 92 ± 16. Although the samples of those who completed the Psychoeducational Group Questionnaire and Press Ganey surveys are not completely matched and the scales on the two measures are different, the Psychoeducational Research Group appears to be a particularly well-liked experience relative to general group programming.

As per the authors’ experience, this new method of administering psychoeducation and cognitive restructuring has been largely positively received. Patients have expressed great interest in and appreciation for this group, with some stating “this [information] gives me hope,” or “I want to make sure this [medical complication] doesn’t happen to me.” Others have suggested this group “helped me clearly see why I have to eat,” can serve as a “nice break” from meal exposures, and is “an interesting new way to help me work against the eating disorder.” Positive preliminary objective and anecdotal findings suggest that further investigation of the group’s usefulness is warranted.

## Discussion

Patients with AN or BN often struggle with intense emotions and distorted beliefs that can promote disordered eating and treatment interfering behaviors. Furthermore, a lack of trust in treatment can result in a poor therapeutic alliance [21, 22], while patients’ anosognosia, disordered thoughts about eating and shape and weight or cognitive inflexibility can inhibit treatment adherence [5, 10, 23]. To help address these challenges, our Psychoeducational Research Group is designed to enhance patients’ understanding of valuable information directly from the scientific literature about eating disorders that may otherwise be unavailable to them outside of treatment.

Rather than using more traditional and conversational Socratic questioning techniques, deferring to and explaining the scientific literature may be a more fruitful method of conducting cognitive restructuring in eating disorders, particularly when taking into account anosognosia and cognitive inflexibility. If a person with anosognosia, for instance, comes to understand the primary scientific literature regarding the negative impact of eating disorders on the body, it becomes difficult to maintain full belief that severe caloric restriction is healthy and could not possibly be responsible for bodily harm. By having a physical scientific paper in hand, group discussion is concrete and focused, thereby removing the opportunity for the person with the eating disorder to dismiss psychoeducational facts the provider is presenting as opinion (e.g. “it’s your opinion that caloric restriction is unhealthy and we can just agree to disagree”). In short, a proposed mechanism of action is the concretizing of group information and discussion, making the cognitive restructuring aspect of the intervention more palatable to the cognitive processing style of individuals with eating disorders.

Another potential mechanism of action could be that exposure to scientific information about the benefits of treatment and recovery may improve outcome expectancies*,* or beliefs about the consequences of engaging in care [24], particularly considering that patients have cited that engaging in the group fosters hope. Research suggests that patients with more positive outcome expectations have enhanced therapeutic alliances and more adaptive treatment outcomes [24, 25].

Based on our preliminary analysis, the group is both helpful and palatable to the patients. It appears patients like this group and would recommend it to others receiving eating disorder treatment. Patients also expressed their group participation was beneficial with regards to challenging eating disorder thoughts, improving motivation for eating behavior change, and working towards treatment goals. When equipped with concrete scientific evidence, this cost-effective intervention may help empower patients to recognize the severity of their illness, challenge and restructure disordered thoughts, refrain from maladaptive behaviors, and understand the logic behind their care.

By educating patients with primary literature, this group can also increase general awareness of research efforts and interest in research participation. Our sister institution, the Columbia Center for Eating Disorders, offers a similar research group to inpatients that emphasizes the importance of scientific research in the treatment of eating disorders. This group’s potential to spark patients’ interests in research initiatives may help expedite the rate at which clinical research is able to improve and be incorporated into clinical care.

### Limitations

This preliminary investigation has certain limitations. First, there is no control group with which to compare our results. Ideally, future research should follow a randomized controlled design so that the impact of the Psychoeducational Research Group can be evaluated relative to a control condition. Second, our results may not be generalizable due to our small sample size of largely Caucasian females. It remains to be determined whether our findings apply to larger samples of male and minority patient populations, as well as to those individuals with eating disorders who are receiving a less acute level of care than inpatient or partial hospitalization. Further, it is possible that the smaller number of patients who participated in the Psychoeducational Research Group more favorably rated the overall inpatient and partial hospitalization experiences. Finally, our study’s assessment, although it may have face validity, does not have established psychometric properties. Further investigation is needed to determine if responses on this assessment translate into patient’s actual cognitive and behavioral changes.

### Future directions

While anecdotal and self-report results are promising, additional research may help elucidate the efficacy of this initiative and how exactly patients utilize this information. As part of a randomized, controlled trial, patients of different genders, ethnicities, and socioeconomic strata should be assessed before and after the group through measurements of their knowledge of eating disorders [26] and overall levels of eating disorder pathology [27]. Evaluating eating disorder psychopathology and symptom frequency (via the Eating Disorder Examination; [28]) before and after treatment among patients who engage in the group and those who receive more traditional psychoeducation may also help clarify the group’s effectiveness. Assessing initial outcome expectancies for treatment among patients in either group should also be explored to help shed light on this novel group’s potential mechanism of action. Furthermore, conducting routine interviews with patients to determine if there are circumstances under which they are able to use this information may help differentiate the effects of evidence-based treatment and this group when they are received in conjunction. Finally, follow-up assessments investigating symptom frequency and severity should be conducted to clarify whether this intervention has any long-term effect on eating disorder course.

## Conclusions

In conclusion, we encourage other eating disorder programs to consider implementing this new form of scientifically-driven, patient-centered psychoeducation. Teaching patients about primary literature in an ongoing, structured group could serve as a useful adjunct to existing therapies and may even enhance adherence to care in patients with illnesses that are notoriously difficult to treat.

## Abbreviations

AN: Anorexia nervosa
BN: Bulimia nervosa
